# Transcriptome Analysis of Tumor-Infiltrating Lymphocytes Identifies NK Cell Gene Signatures Associated With Lymphocyte Infiltration and Survival in Soft Tissue Sarcomas

**DOI:** 10.3389/fimmu.2022.893177

**Published:** 2022-07-07

**Authors:** Sean J. Judge, Joshua D. Bloomstein, Cyrus J. Sholevar, Morgan A. Darrow, Kevin M. Stoffel, Logan V. Vick, Cordelia Dunai, Sylvia M. Cruz, Aryana M. Razmara, Arta M. Monjazeb, Robert B. Rebhun, William J. Murphy, Robert J. Canter

**Affiliations:** ^1^ Division of Surgical Oncology, Department of Surgery, University of California, Davis, Sacramento, CA, United States; ^2^ Department of Pathology and Laboratory Medicine, University of California, Davis, Sacramento, CA, United States; ^3^ Department of Dermatology, University of California, Davis, Sacramento, CA, United States; ^4^ Department of Radiation Oncology, University of California, Davis, Sacramento, CA, United States; ^5^ Center for Companion Animal Health, Surgical and Radiological Sciences, School of Veterinary Medicine, University of California, Davis, Davis, CA, United States; ^6^ Division of Hematology and Oncology, Department of Medicine, University of California, Davis, Sacramento, CA, United States

**Keywords:** soft tissue sarcoma (STS), NK cells, TIL (tumor infiltrating lymphocytes), immune infiltrate, T cells, immuno oncology

## Abstract

**Purpose:**

Clinical successes using current T-cell based immunotherapies have been limited in soft tissue sarcomas (STS), while pre-clinical studies have shown evidence of natural killer (NK) cell activity. Since tumor immune infiltration, especially tumor-infiltrating lymphocytes, is associated with improved survival in most solid tumors, we sought to evaluate the gene expression profile of tumor and blood NK and T cells, as well as tumor cells, with the goal of identifying potential novel immune targets in STS.

**Experimental Design:**

Using fluorescence-activated cell sorting, we isolated blood and tumor-infiltrating CD3^-^CD56^+^ NK and CD3^+^ T cells and CD45^-^ viable tumor cells from STS patients undergoing surgery. We then evaluated differential gene expression (DGE) of these purified populations with RNA sequencing analysis. To evaluate survival differences and validate primary DGE results, we also queried The Cancer Genome Atlas (TCGA) database to compare outcomes stratified by bulk gene expression.

**Results:**

Sorted intra-tumoral CD3^+^ T cells showed significant upregulation of established activating (CD137) and inhibitory genes (TIM-3) compared to circulating T cells. In contrast, intra-tumoral NK cells did not exhibit upregulation of canonical cytotoxic genes (IFNG, GZMB), but rather significant DGE in mitogen signaling (DUSP4) and metabolic function (SMPD3, SLC7A5). Tumors with higher NK and T cell infiltration exhibited significantly increased expression of the pro-inflammatory receptor TLR4 in sorted CD45^-^ tumor cells. TCGA analysis revealed that tumors with high TLR4 expression (*P* = 0.03) and low expression of STMN1 involved in microtubule polymerization (*P* < 0.001) were associated with significantly improved survival.

**Conclusions:**

Unlike T cells, which demonstrate significant DGE consistent with upregulation of both activating and inhibiting receptors in tumor-infiltrating subsets, NK cells appear to have more stable gene expression between blood and tumor subsets, with alterations restricted primarily to metabolic pathways. Increased immune cell infiltration and improved survival were positively correlated with TLR4 expression and inversely correlated with STMN1 expression within tumors, suggesting possible novel therapeutic targets for immunotherapy in STS.

## Introduction

Soft tissue sarcomas (STS) comprise a diverse group of tumors of mesenchymal differentiation that together account for approximately 13,000 cancer cases per year in the United States (1). Significant challenges exist in the care of these patients, and patients with non-metastatic, locally advanced high grade tumors have a risk of distant recurrence and death as high as 50% within 5 years of diagnosis (2). Once metastatic, median survival is approximately 1 year, and 5-year survival for these patients is very poor at approximately 15% (1, 3, 4). Despite increased knowledge regarding the molecular biology and genetics of STS, few significant advances have been made in the treatment of these patients and novel therapeutic approaches are needed, including immunotherapy. Response rates with standard checkpoint blockade immunotherapy (using anti-PD-1, PD-L1, and CTLA-4 monoclonal antibodies) have been modest for STS, and as a result, these therapies remain unapproved for this family of diseases (5). Data from our lab and others have shown that natural killer (NK) cells home to STS tumors (especially when T cell infiltration is low) and that NK cells are active against STS in preclinical sarcoma models (6, 7). Overall, NK-based strategies appear promising in STS, but successful translation of NK cell therapeutics, which capitalize on endogenous NK tumor-targeting or adoptive therapy, have yet to be realized in most solid tumors (8).

Barriers to the successful translation of NK therapies, especially for solid tumors, include homing to the solid tumor microenvironment (TME), persistence within the TME, and development of NK dysfunction within the TME from immunosuppressive metabolic signals and cellular constituents (9). Efforts to overcome these barriers include novel mechanisms to augment the function of tumor-infiltrating lymphocytes and mechanisms to promote immune cell infiltration. While much progress has been made in the field of T-cell biology, a better understanding of extrinsic or tumor-specific drivers of NK cell dysfunction in the TME remains to be elucidated, especially in STS.

Therefore, the goal of our study was to assess purified NK and T cells from STS tumors and the blood to determine if gene signatures could be identified which distinguish intra-tumoral NK and T cells from those in the blood, which associate with greater TIL infiltration, and which may be correlated with survival.

## Methods

### Collection and Preparation of Human Tissues

The collection of matched whole blood and tumor specimens was approved by the IRB at the University of California, Davis (Protocol # 218204-9). Human peripheral blood mononuclear cells (PBMCs) were isolated from whole blood using a density gradient centrifugation (Lymphocyte Separation Medium, Corning Life Sciences), followed by red blood cell lysis, as described previously (10). Tumor tissue from patients undergoing STS resection was collected in sterile RPMI-1640 (Invitrogen Life Technologies), diluted with PBS and passed through a 70 µm filter, and then subjected to red blood cell lysis.

### Flow Cytometry

Human cells were washed with PBS and staining buffer, and then incubated with Human TruStain Fc receptor blocking solution (BioLegend, #422302) for 15 minutes. Cells were stained with the following fluorochrome-conjugated monoclonal antibodies: CD3-FITC (clone HIT3a, BioLegend), CD56-PE (clone HCD56, BioLegend), CD45-BV510 (clone HI30, BioLegend) and CD8-BV785 (clone RPA-T8, BioLegend). Live/dead staining was performed using Fixable Viability Dye 780 (eBioscience #65-0865-14). Data were acquired using a BD LSRFortessa flow cytometer (Becton Dickinson, San Jose, CA) and analyzed using FlowJo Software (Beckton Dickinson, San Jose, CA). Fluorescence-associated cell sorting (FACS) was performed using a BD inFlux cell sorter (Beckton Dickinson, San Jose, CA). Cells were sorted for blood and intra-tumoral NK cells (live CD45^+^CD3^-^CD56^+^), blood and intra-tumoral T cells (live CD45^+^CD3^+^CD56^-^), and tumor cells (live CD45^-^). Following FACS isolation, cells were cryopreserved and stored in liquid nitrogen for subsequent RNA extraction and sequencing.

### RNA Sequencing of NK and T Cells

Cells were processed for RNA isolation using the Total RNA Purification Plus Micro Kit (Norgen Biotek, Ontario, Canada). RNAseq libraries were prepared by the University of California, Davis, Bioinformatics Core (11). Gene expression profiling was carried out using a 3’-Tag-RNA-Seq protocol. Up to forty-eight libraries were sequenced per lane on an Illumina HiSeq 4000 sequencer (Illumina, San Diego, CA) with single-end 100 base pair reads. Differential gene expression analysis was performed with the *DESeq2* package for R. The Benjamini-Hochberg procedure was used to adjust for False Discovery Rate (FDR) ≤ 0.05.

### Gene Set Enrichment Analysis

Gene Set Enrichment Analysis (GSEA) was performed using WebGestalt (12). The functional database used was Kyoto Encyclopedia of Genes and Genomes (KEGG). Log2 fold-change values comparing tumor NK cells to peripheral NK cells, and tumor T cells to peripheral T cells, for genes with *p* < 0.01 were loaded to WebGestalt. GSEA was run with default parameters.

### TCGA

Clinicodemographic and RNA expression data were retrieved from The Cancer Genome Atlas (TCGA) SARC dataset using the UCSC Xena platform (retrieved July 5, 2021) (13). High and low expression groups were determined using median values, as described previously (7, 14, 15). Survival differences were estimated using Kaplan-Meier analysis with log-rank test. NK and CD8 signatures were generated as previously described (16). Correlations were compared using Spearman correlation test.

### Statistical Analysis

Prism software (GraphPad Software Inc.), R 3.6.1 (R Foundation for Statistical Computing, Vienna, Austria), and Python 3.7.4 (Python Software Foundation, https://python.org/) for plot generation and statistical analysis. Data were expressed as mean ± SEM. Where appropriate, normality of distribution was evaluated using Shapiro-Wilk normality test. Differences between two groups were analyzed using the paired or unpaired Student’s t-test as appropriate for parametric data and the Mann-Whitney U-test or Wilcoxon signed-rank test for non-parametric data. For analysis of three or more groups, one-way analysis of variance (ANOVA) tests was performed with Tukey’s or Dunnett’s post-test as appropriate. Correlations between two variables were calculated using Spearman correlation test. Kaplan-Meier estimates and log-rank test were used to compare survival outcomes between subgroups. *P* ≤ 0.05 was considered statistically significant, and an adjusted *P*-value ≤ 0.05 was considered statistically significant for gene expression analyses.

## Results

### Clinicodemographic Information on Patients Utilized in Prospective Study

**Figure 1:**
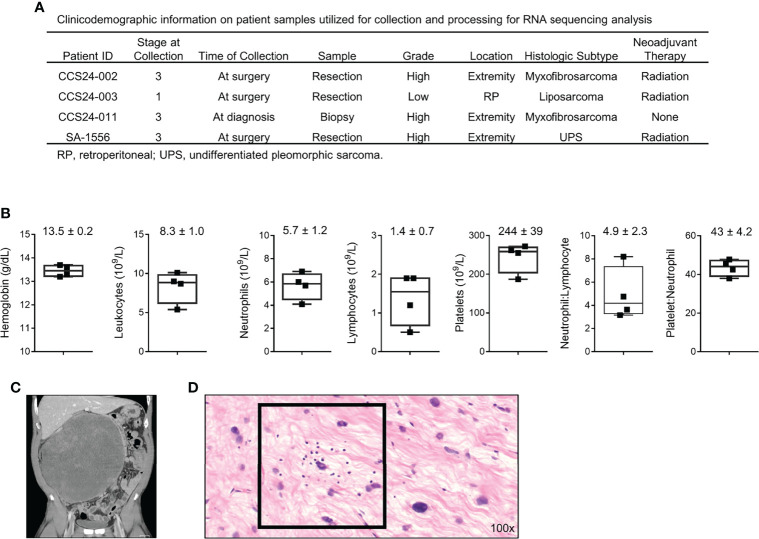
Clinicodemographic information on patients used in this prospective study. ***(*A*)*** Clinical information regarding the four patients analyzed in this cohort. **(B)** Pre-operative peripheral blood laboratory values for each patient. **(C)** Pre-operative computed tomography (CT) imaging showing a locally advanced well-differentiated liposarcoma of the retroperitoneum from patient CCS24-003. **(D)** Photomicrograph of the tumor from patient CCS24-003 showing tumor infiltrating lymphocytes Representative H&E image showing tumor infiltrating lymphocytes (TILs) (H&E, 100x).

We prospectively collected blood and tumor tissue for transcriptomic analysis from four STS patients undergoing surgical resection with curative intent (**Figure 1A**). Peripheral blood was drawn on the day of surgery, and pertinent laboratory values are shown in **Figure 1B**. Neutrophil to lymphocyte (N:L) and platelet to neutrophil (P:N) ratios for the cohort were 4.2 (range 3.2 – 8.2) and 44 (range 38 – 48), respectively. Representative pre-operative cross-sectional imaging is shown for patient CCS24-003 (**Figure 1C**), highlighting the substantial disease burden for this cohort of patients. Representative H&E photomicrograph from tumor CCS24-003 shows infiltrating lymphocytes, which is typically variable in STS (**Figure 1D**) (7, 17, 18).

### Peripheral Blood Immune Cells and Tumor Immune Infiltrate Shows Population Differences in Soft Tissue Sarcoma

**Figure 2:**
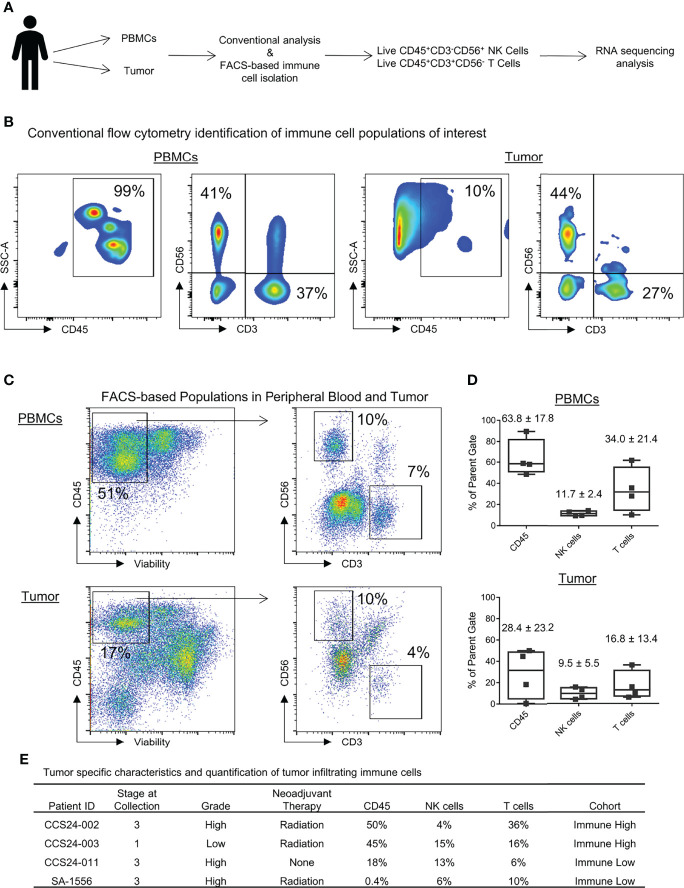
Identification of immune populations and heterogeneity in immune infiltrates within soft tissue sarcomas. ***(*A*)*** Schema for experimental design and identification of pertinent peripheral and tumor-infiltrating immune populations. **(B)** Identification of peripheral and tumor-infiltrating NK and T cells by conventional flow cytometry. **(C)** Gating strategy to identify and isolate immune cells of interest by FACS for RNA sequencing analysis. **(D)** Quantification of CD45^+^, NK, and T cells shown as percent of parent populations as determined by FACS gating. **(E)** The tumor specific characteristics and quantification of tumor infiltrating immune cells for each patient analyzed in this study (shown as percent of parent population).

We then isolated blood and tumor-infiltrating CD3^-^CD56^+^ NK and CD3^+^ CD56^-^ T cells for flow phenotyping and RNA sequencing analysis. The schema for our experimental design is shown in **Figure 2A**. Results from conventional flow cytometry are shown in **Figure 2B**. Within the peripheral blood, as expected following mononuclear cell isolation, the vast majority of live cells were CD45^+^ leukocytes. Although CD3^+^ T cells standardly outnumbered NK cells, one patient notably showed a near equivalence of NK and T cells (**Figure 2B**). In contrast to the blood, CD45^+^ cells represented only approximately 10% of live cells within tumors, highlighting the generally low immune cell infiltrate in most STS (19). Blood and tumor specimens were also processed for fluorescence-activated cell sorting (FACS) of CD45^+^CD3^-^CD56^+^ NK cells, CD45^+^CD3^+^CD56^-^ T cells, and CD45^-^ tumor cells, with representative sorting gates shown in **Figure 2C**. Using the FACS gating schema and percent of parent population, relative percentage of leukocytes, NK cells and T cells for both peripheral blood and tumor are shown in **Figure 2D**. Within peripheral blood NK cells represented 11.7 ± 2.4% of viable CD45^+^ cells while they represented 9.5 ± 5.5% of live leukocytes in the tumor. In contrast, CD3^+^CD56^-^ T cells represented 34.0 ± 21.4% and 16.8 ± 13.4% of live leukocytes in blood and tumor, respectively (**Figure 2D**). The quantification of tumor-infiltrating immune cells and corresponding tumor stage, grade, and neoadjuvant therapies are shown in **Figure 2E**. There was no apparent correlation between the receipt of neoadjuvant radiation therapy (CCS24-002, CCS24-003, SA-1556) and increased CD45^+^ immune cell infiltration. Similar observations were made for stage and tumor grade.

### Transcriptomic Analysis of Peripheral and Tumor-Infiltrating Immune Cells Highlights Distinct Changes in Gene Expression Patterns Between NK and T Cells in Soft Tissue Sarcomas

**Figure 3:**
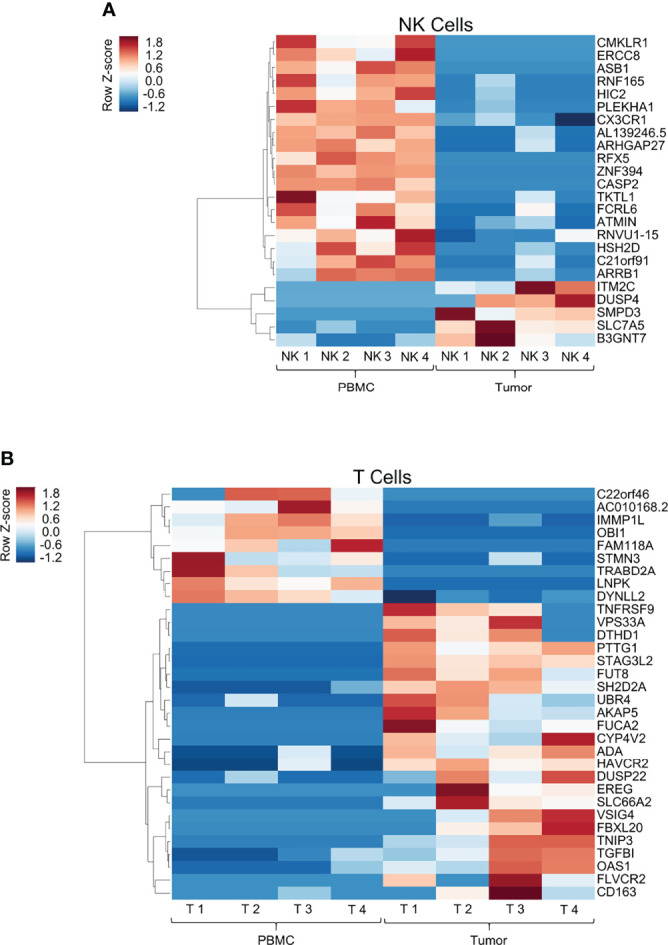
Transcriptomic analysis of peripheral and tumor-infiltrating immune cells highlights distinct changes in gene expression patterns between NK and T cells in soft tissue sarcomas. Heatmap showing log2 cpm (counts per million) of differentially expressed genes from comparison of peripheral and tumor-infiltrating NK cells (**A**, top panel) and T cells (**B**, bottom panel) isolated from patients with soft tissue sarcomas undergoing definitive surgery. Genes were arranged by hierarchical clustering using correlation distance and average linkage. **(A)** 24 genes had significant DGE within intra-tumoral NK cells compared to blood, of which only 20% were upregulated within the tumor. **(B)** In contrast, 32 genes had significant DGE within intra-tumoral T cells compared to blood, of which 72% exhibited upregulated expression within the tumor.

There is currently a thorough and evolving understanding of the genetic and epigenetic changes in tumor-infiltrating T cells which distinguish them from naïve, circulating T cells that populate the peripheral blood (20, 21). In addition, the diversity of T cell populations within the solid tumor microenvironment (TME) is increasingly being recognized and characterized (22). Importantly, a similar understanding is not as well established for tumor-infiltrating NK cells, but significant advances have been made (23). Given this background, we set out to analyze the transcriptomic profile of tumor-infiltrating NK cells in STS compared to peripheral NK cells, particularly in the context of a side-by-side analysis of differential gene expression (DGE) from matched tumor-infiltrating and peripheral T cells. Genes with significant differential expression between the blood and the tumor for NK cells (**Figure 3A**) and T cells (**Figure 3B**) are shown as heatmaps in **Figure 3**. Globally, comparing expression in the blood to the tumor compartment, we detected 24 genes in NK cells and 32 genes in T cells with significant differential expression, likely representing true positive differences given the false discovery rate we employed in our statistical analysis. Within these differentially expressed genes, we identified 19 genes which were significantly downregulated and 5 which were significantly upregulated by NK cells in the tumor. In contrast, we identified nine genes which were significantly downregulated and 23 genes which were significantly upregulated by T cells in the tumor, compared to expression by the same cell populations in the blood.

Tumor-infiltrating T cells significantly upregulated well-established co-stimulatory and inhibitory receptors TNFRSF9 (CD137, 4-1BB) and HAVCR2 (TIM-3). In contrast, intra-tumoral NK cells did not follow a comparable DGE pattern. Instead, as shown in **Figure 3A**, we observed significant upregulation of genes utilized in mitogen signaling and cell cycle processes (DUSP4, SLC7A5). Moreover, we did not observe transcriptional upregulation of any established checkpoint markers (PD-1, TIGIT, CTLA-4, TIM-3, LAG-3, etc.) in tumor NK cells, though this could be a function of measuring active RNA transcriptional differences by RNA sequencing as opposed to protein expression by other laboratory techniques. To ensure our sorting purity, we compared tumor-infiltrating NK and T cell gene expression, highlighting unequivocal differences in DGE of canonical NK and T cell genes among the respective subsets, specifically NCAM1 and IL2Rb in NK cells, and CD3, CD8 and CD28 in T cells (**Supplemental Figure 1**).

Gene Set Enrichment Analysis (GSEA) was performed with the NK and T cell populations comparing the tumor-infiltrating cells to the peripheral counterpart and is shown in **Supplemental Figure 2**. The most prominent pathways detected are shown for NK cells (**Supplemental Figure 2A**) and T cells (**Supplemental Figure 2B**).

### Tumor-Intrinsic Transcriptomic Differences in Immune High vs Immune Low Soft Tissue Sarcomas

**Figure 4:**
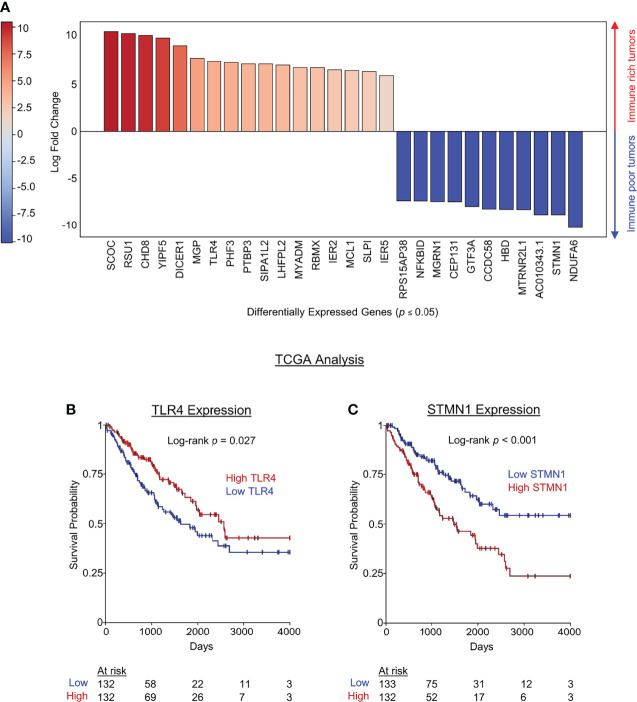
Tumor-intrinsic transcriptomic differences in immune high vs immune low soft tissue sarcomas. Tumors samples were segregated based on immune infiltrate (above or below median percent CD45^+^ infiltrate), and differential gene expression was performed using live CD45^-^ tumor cells. **(A)** Genes in red were significantly upregulated in high immune infiltrate tumors while genes in blue were significantly downregulated in high infiltrate tumors. **(B)** TCGA data were then used to analyze survival differences from publicly available data based on DGE identified in our cohort which distinguished immune infiltrate high and low STS tumors. The pro-inflammatory pattern recognition receptor TLR4 was significantly increased in our series of immune rich tumors and associated with improved overall survival by Kaplan-Meier estimate of the TCGA data. **(C)** STMN1 was significantly increased in immune poor tumors and high STMN1 expression was associated with decreased overall survival the TCGA dataset.

Tumor mutational burden (TMB) has been linked to neoantigen load and response to checkpoint blockade in solid tumor patients (24–26), but tumor-specific transcriptomic differences underlying immune cell infiltration and more favorable survival outcomes among cancer patients are currently unknown. We therefore set out to investigate tumor-intrinsic transcriptomic differences between STS tumors with high and low leukocyte infiltration (live CD45^+^ cells). We compared RNA expression in tumors with above median leukocyte infiltration (CCS24-002 and CCD24-003) to those with below median leukocyte infiltration (CCS24-011 and SA1556). DGE analysis identified 28 tumor-cell expressed genes which were significantly different (*p* < 0.05) between immune high and low tumors. These genes are shown in **Figure 4A**. The 17 genes in red had significantly increased expression in the immune rich tumors, while the 11 genes in blue were significantly increased in the immune poor tumors. These genes were then investigated for associations between expression and overall survival using The Cancer Genome Atlas (TCGA). The TCGA-SARC dataset was queried using the UCSC Xena platform to evaluate differences in tumor gene expression and clinical outcomes in 265 adults with varying histologic subtypes of STS. We observed that high tumor expression of TLR4 was associated with improved overall survival (*p* = 0.027) (**Figure 4B**), whereas low tumor expression of STMN1 was associated with improved overall survival (*p* < 0.001) (**Figure 4C**), both by Kaplan-Meier survival analysis. No other genes queried showed statistically significant survival differences within the TCGA-SARC dataset. Using the previously described NK and CD8 gene expression signatures (16), correlations were made with TLR4 and STMN1 expression (**Supplemental Figure 3**). For the NK signature, there was a strong direct correlation with TLR4 (r = 0.6, *p* < 0.0001) and indirect correlation with STMN1 (r = -0.2, *p* < 0.001) (**Supplemental Figure 3A**). As for the CD8 signature, there was also a strong direct correlation with TLR4 expression (r = 0.5, *p* < 0.0001), but not with STMN1 expression (**Supplemental Figure 3B**). These results show the correlation with NK and CD8 T cells and TLR4 expression within the tumor, though the TLR4-expressing cell type cannot be clearly delineated from this dataset.

## Discussion

There is a growing appreciation that tumor-infiltrating immune cells are distinct from their peripheral, circulating counterparts. This is especially true for T cells, which must undergo a series of pivotal phenotypic and functional changes to transition from a circulating naïve T cell, to a tumor-infiltrating effector memory T cell (27). Although intra-tumoral T cells are clearly associated with superior oncologic outcomes, it is also established that tumor-infiltrating T cells are characterized by a spectrum of dysfunction which frequently permits immune escape and tumor progression (28–30). Efforts to target and reverse the exhausted subpopulation of intra-tumoral memory T cells have revolutionized cancer therapy. However, these paradigms have been less well characterized for intra-tumoral NK cells, in part because NK cells are less abundant in solid tumors, delineation of specific subsets has been less definitive, and therapeutic exploitation of NK cells for solid tumors has been difficult to realize to date (9, 31, 32). Additionally, the tumor-specific gene expression that may drive immune cell infiltration is an unanswered question in cancer biology and immunotherapy. Here, as with T cells, we show that intra-tumoral NK cells show a distinct transcriptional profile compared to their circulating counterparts. However, unlike T cells, the differences identified in NK cell DGE do not involve well-established checkpoint markers or cell surface receptors, but rather appear to be genes involved in basic metabolic processes. This observation would suggest that either the metabolic/environmental conditions of the TME are affecting NK cell gene expression to a greater extent than exposure to tumor targets or that NK effector-target interactions are causing alterations in metabolic gene expression pathways more than those involved in activation and immunoregulation, at least in STS. While we are unable to answer this question with the current data, our results do intriguingly show that gene expression alterations within intra-tumoral NK cells are distinct from those of intra-tumoral T cells, suggesting (somewhat provocatively) that strategies and techniques to augment NK cell function for greater anti-tumor effects in STS and perhaps other solid tumors will be different (8, 10, 33, 34).

One specific gene identified as upregulated in the tumor infiltrating NK population was SLC7A5. Also known as Large Amino Acid Transporter (LAT) 1, SLC7A5 is a critical component of a transporter complex involved in essential amino acid transport (35). Prior work has shown that SLC7A5 is critical for T lymphocyte clonal expansion and effector differentiation following TCR engagement (36). Extrapolating from this observation, it is likely that SLC7A5 is critical in other cytotoxic leukocytes, including NK cells. In fact, recent data from Almutairi et al. showed that several cytokines, including IL-12 and IL-18, led to the upregulation of amino acid transporters on NK cells, specifically including SLC7A5 (37). It is therefore likely that SLC7A5 is critical to NK function and represents an appropriate response for intra-tumoral NK cells.

Our results also identified a series of tumor-intrinsic genes which were differentially enriched in immune-rich compared to immune-poor tumors. Notably, the tumors with high immune cell infiltration had significantly increased expression of TLR4, a member of the Toll-like receptor family responsible for detecting lipopolysaccharide (LPS), a key component of Gram-negative bacteria (38). Apart from their expression on various immune cells, TLRs (including TLR4) are known to be expressed on various tumor cell lines and primary tumors from various sites within the body (39). Signaling through TLR4 induces various cytokines that have been associated with both tumor-promoting and tumor-resisting effects (39). Several studies have also shown a beneficial effect of TLR4 agonists *in vivo* to promote anti-tumor responses (40), with a recent publication highlighting the combination of PD-1 blockade and TLR4 stimulation with brucella lumazine synthase to increase the immune infiltrate and anti-tumor effects in B16 melanoma pre-clinical models (41). A recently completed phase I clinical trial of intratumoral TLR4 agonist G100 in ten patients with advanced Merkel cell carcinoma also showed promising results with partial and complete responders during a median follow up of 33 months, in conjunction with histologic evidence of increased cytotoxic immune cell infiltration (42). Our data are in alignment with these findings and suggest that increased tumor expression of TLR4 in STS may therefore represent a mechanism for driving immune cell infiltration, especially cytotoxic lymphocytes, and therapies to target this pathway may prove promising.

In contrast to TLR4, leukocyte-poor tumors in our cohort showed increased expression of a distinct set of genes. In particular, STMN1 (Stathmin1), a regulatory protein in cytoskeletal and microtubular processes, was significantly upregulated in immune-poor tumors and simultaneously associated with worse survival on analysis of the TCGA-SARC database. Similarly, a recent meta-analysis observed that high expression of Stathmin1 was associated with worse survival in diverse solid cancers (43). Apart from its role in cell division and mitosis, the role of STMN1 in limiting immune cell infiltration remains unclear, and the evidence we provide in support of this negative association between STMN1 expression, low immune infiltrate, and worse survival outcomes is worthy of further investigation.

Although our study is clearly limited by small sample size, a strength of this work is our focus of high throughput RNA sequencing on purified, flow-sorted NK and T cell populations from matched peripheral blood and tumor tissues of STS patients. RNA sequencing remains a powerful tool to evaluate the cellular and molecular factors which shape the TME, and strategies which rely on bulk RNA sequencing of tumors are unable to detect the contribution of specific immune subsets, whereas single cell RNA sequencing techniques can be less quantitatively rigorous than those of bulk RNA sequencing (44). In addition, our ability to validate our intra-tumoral NK and T cell sequencing data with data from the TCGA reinforces the clinical relevance of this work.

Despite these relevant strengths, it is nevertheless important to acknowledge the weaknesses of this study. Pre-clinical studies are needed to determine the influence of these immune-specific and tumor-specific genes on driving tumor infiltration and survival differences and dissect the direct versus indirect effects of these genes on immune infiltration, cancer growth, and survival. Moreover, as noted above, the small number of patients analyzed in this cohort introduces risks of bias and skewing which undermine the ability to derive concrete conclusions from this work. Nonetheless, we maintain that this hypothesis-generating work is a contribution to both STS cancer biology and NK immunobiology studies, especially given the current limitations in successful immunotherapeutic strategies for patients with this family of frequently aggressive diseases (45). An additional weakness is the use of CD3 as the sole positive marker for T cells. This may impact the results since specific T cell subsets were not analyzed and can have immunosuppressive (Treg) or cytotoxic (CD8^+^) effects. Further analysis of T cell subsets is expected to improve the translation of these findings, especially with respect to the similarities and differences between tumor-infiltrating NK and CD8+ T cells.

In summary, our data suggest that intra-tumoral NK cells exhibit a unique transcriptional profile which is distinct from both their circulating NK counterparts as well as from intra-tumoral T cells. In addition, we identified different transcriptional profiles in the tumor cells of STS tumors with high versus low immune infiltrates. Therefore, we conclude that further investigation into the role of NK-specific metabolic processes and tumor expression of TLR4 and STMN1 is indicated to increase understanding of the genes that drive immune cell infiltration into STS and how expression of these genes can be targeted to improve immunotherapy responses in STS.

## Data Availability Statement

The datasets presented in this study can be found in online repositories. The names of the repository/repositories and accession number(s) can be found below: NCBI GEO, accession no: GSE205492.

## Ethics Statement

All experiments were approved by the Institutional Review Board at the University of California, Davis, School of Medicine. The patients/participants provided their written informed consent to participate in this study.

## Author Contributions

SJ and RC designed the study. SJ, JB, and MD conducted the experiments and collected the data. KS, CD, and LV provided technical assistance. SJ and RC analyzed the data. SJ and RC wrote the manuscript. All authors, including CS, SC, AR, AM, RR, and WM provided critical reviews of the manuscript. All authors contributed to the article and approved the submitted version.

## Funding

This work was supported in part by National Institutes of Health/National Cancer Institute grant U01 CA224166-01 (RC, RR) and R03CA252793 (RC). This work was also supported in part by funds from the UC Davis Comprehensive Cancer Center and the University of California Davis Flow Cytometry Shared Resource Laboratory with funding from the NCI P30 CA093373 (Cancer Center), S10 OD018223 (Astrios Cell Sorter), and S10 RR 026825 (Fortessa Cytometer) grants, with technical assistance from Ms. Bridget McLaughlin and Mr. Jonathan Van Dyke. Specimens were provided by the UC Davis Pathology Biorepository which is jointly funded by the UC Davis Comprehensive Cancer Support Grant (CCSG) awarded by the National Cancer Institute (NCI P30 CA093373) and the UC Davis Department of Pathology and Laboratory Medicine. The sequencing was carried out at the DNA Technologies and Expression Analysis Cores at the UC Davis Genome Center, supported by NIH Shared Instrumentation Grant 1S10OD010786-01, with technical assistance from Ms. Emily Kumimoto, Ms. Claire Barron Goldman, and Dr. Lutz Froenicke.

## Conflict of Interest

The authors declare that the research was conducted in the absence of any commercial or financial relationships that could be construed as a potential conflict of interest.

## Publisher’s Note

All claims expressed in this article are solely those of the authors and do not necessarily represent those of their affiliated organizations, or those of the publisher, the editors and the reviewers. Any product that may be evaluated in this article, or claim that may be made by its manufacturer, is not guaranteed or endorsed by the publisher.

## Supplementary Material

Supplementary Figure 1 Transcriptomic analysis of tumor-infiltrating NK and T cells confirms sorting purity and identifies canonical gene expression profile of the immune cell types. Heatmap showing log2 cpm (counts per million) of differentially expressed genes from comparison of tumor-infiltrating NK cells (left side) and T cells (right side) isolated from patients with soft tissue sarcomas undergoing definitive surgery.

Supplementary Figure 2
*Gene set enrichment analysis (GSEA)of immune cells in patients with soft tissue sarcoma.* GSEA was performed on purified NK and T cells, comparing the tumor-infiltrating cells to the peripheral counterpart. The most represented pathways are shown for NK cells **(A)** and T cells **(B)**. Significantly enriched pathways were only detected in T cells, shown in solid blue and orange (FDR ≤ 0.05).

Supplementary Figure 3 Association between NK and CD8 gene expression signatures and expression of TLR and STMN1. The TCGA SARC dataset was analyzed to identify a correlation between immune cells present within the tumor and expression of TLR4 and STMN1. **(A)** The NK gene expression signature showed a significant positive correlation with TLR4 (r = 0.6, *p* < 0.0001) and a significant negative correlation with STMN1 (r = -0.2, *p* < 0.001). **(B)** The CD8 gene expression signature also showed a significant positive correlation with TLR4 (r = 0.5, *p* < 0.0001), but an insignificant negative correlation with STMN1 (r = -0.1, *p* = 0.07).
